# Aβ42-oligomer Interacting Peptide (AIP) neutralizes toxic amyloid-β42 species and protects synaptic structure and function

**DOI:** 10.1038/srep15410

**Published:** 2015-10-29

**Authors:** Christian Barucker, Heiko J. Bittner, Philip K.-Y. Chang, Scott Cameron, Mark A. Hancock, Filip Liebsch, Shireen Hossain, Anja Harmeier, Hunter Shaw, François M. Charron, Manuel Gensler, Paul Dembny, Wei Zhuang, Dietmar Schmitz, Jürgen P. Rabe, Yong Rao, Rudi Lurz, Peter W. Hildebrand, R. Anne McKinney, Gerhard Multhaup

**Affiliations:** 1 Department of Pharmacology and Therapeutics, Faculty of Medicine, McGill University, Montreal, QC, Canada; 2 Institute of Medical Physics and Biophysics, Charite-Universitätsmedizin, Germany; 3 Department of Neurology and Neurosurgery, Montreal, McGill Centre for Research in Neuroscience, Canada; 4 Institut für Chemie und Biochemie, Freie Universität Berlin, Germany; 5 Department of Physics & IRIS Adlershof, Humboldt-Universität zu Berlin, Germany; 6 Neurowissenschaftliches Forschungszentrum, Charite-Universitätsmedizin, German Center for Neurodegenerative Diseases (DZNE), Berlin, Germany; 7 Max-Planck-Institute for Molecular Genetics, Berlin, Germany

## Abstract

The amyloid-β42 (Aβ42) peptide is believed to be the main culprit in the pathogenesis of Alzheimer disease (AD), impairing synaptic function and initiating neuronal degeneration. Soluble Aβ42 oligomers are highly toxic and contribute to progressive neuronal dysfunction, loss of synaptic spine density, and affect long-term potentiation (LTP). We have characterized a short, L-amino acid Aβ-oligomer Interacting Peptide (AIP) that targets a relatively well-defined population of low-n Aβ42 oligomers, rather than simply inhibiting the aggregation of Aβ monomers into oligomers. Our data show that AIP diminishes the loss of Aβ42-induced synaptic spine density and rescues LTP in organotypic hippocampal slice cultures. Notably, the AIP enantiomer (comprised of D-amino acids) attenuated the rough-eye phenotype in a transgenic Aβ42 fly model and significantly improved the function of photoreceptors of these flies in electroretinography tests. Overall, our results indicate that specifically “trapping” low-n oligomers provides a novel strategy for toxic Aβ42-oligomer recognition and removal.

The amyloid-β42 (Aβ42) peptide is considered as the main culprit in the pathogenesis of Alzheimer disease (AD)^1^, postulated to impair synaptic function and initiate neuronal degeneration^2^,^3^. Though the evidence for a central role of Aβ in the pathogenesis is very strong^4^, other models support a modulatory function for low Aβ concentrations on neurotransmission and memory^5^,^6^.

Similar to other amyloid diseases, metastable oligomers and non-fibrillar amyloid intermediates can cause proteotoxicity in AD^7^. Intracellular tau, intracellular and extracellular Aβ can lead to cell death *in vivo* and *in vitro*^8^,^9^,^10^,^11^,^12^. Since soluble species of Aβ42 are more strongly correlated with disease symptoms compared to amyloid fibrils^13^, these toxic oligomers are believed to underlie losses in hippocampus synapses (occurs early in the disease process) and correlate with the degree of cognitive impairment in AD patients^14^. Naturally secreted Aβ42 oligomers can disrupt cellular models of learning and memory, hippocampal long-term potentiation (LTP) in acute slices and *in vivo*, and impair the memory of a complex learned behavior in rats^15^. Usually, such studies have been conducted with higher concentrations while physiological concentrations are rather in the picomolar range and may have even beneficial effects^6^. Nevertheless, *in vivo*, Aβ is always a mixture of various aggregates, and a defined biological concentration of a specific oligomeric conformation has not been determined yet^4^.

Soluble low-n oligomers (i.e. tetramers and hexamers) of Aβ42 are neurotoxic^15^,^16^ and ascribed to promote the progressive loss of dendritic spines and glutamatergic synapses^17^,^18^. It is plausible that the generation of toxic Aβ oligomers may be the result of newly formed aggregates, and/or the depolymerization of amyloid plaques. In either case, the cellular mode-of-action is not fully understood.

In the present study, we performed a peptide-based anti-amyloid approach, which differed from previously reported anti-oligomer strategies. A diverse array of biomolecules have already been identified which possess the ability to prevent amyloid fibril formation both *in vitro* and *in vivo*—large proteins such as molecular chaperones^19^, to small molecules such as flavonoids^20^, polyphenols^21^, hydroxyquinoline derivatives^22^ and peptide-based inhibitors^23^,^24^,^25^,^26^,^27^. Preferably, the compound should neither be a dissociation catalyst nor a monomer competitor but rather interact with potentially toxic Aβ oligomers to inhibit the conversion of low-n Aβ42 oligomers into mature amyloid fibrils. Here, we demonstrate that an 8-residue long peptide (RGTFEGKF), initially designed on the framework GxFxGxF to disrupt sheet-to-sheet packings of Aβ40 fibrils^27^ can specifically target low-n Aβ42 oligomers (mainly tetramers and hexamers), rather than simply inhibit Aβ aggregation. Compared to earlier anti-amyloid oligomer strategies, AIP is unique in that this Aβ-oligomer Interacting Peptide (AIP) can “trap” toxic amyloid oligomers—i.e. inhibit the conversion of low-n Aβ42 oligomers into mature amyloid fibrils, neutralize their toxicity and prevent the growth of Aβ oligomers into larger assemblies.

## Results

### Characterization of AIP on Aβ42 self-aggregation

**Figure 1:**
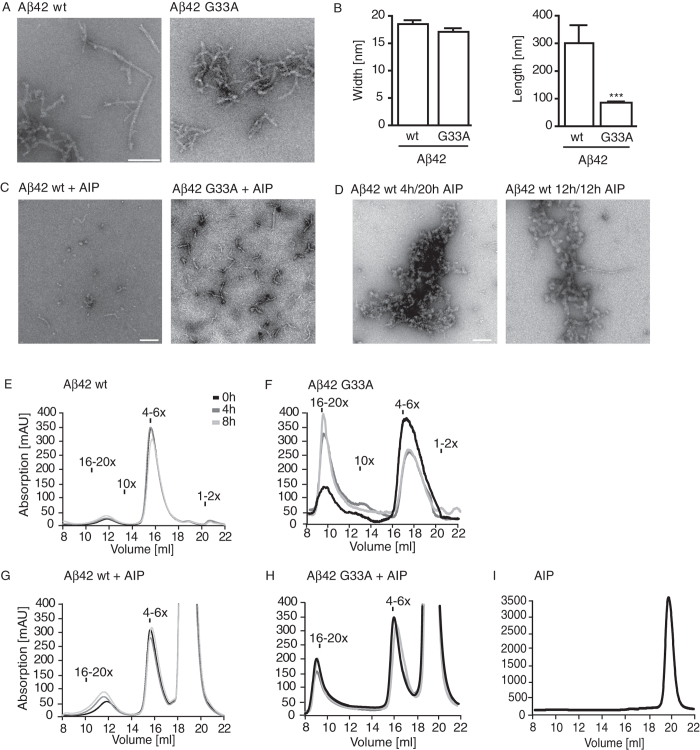
Aggregation studies of Aβ42 wt and Aβ42 G33A peptides. (**A**) TEM micrographs were acquired after 24 hours incubation. Aβ42 wt aggregated to mature fibrils whereas Aβ42 G33A solely formed globular oligomers and protofibrilar structures. Scale bar = 200 nm. The graphs in (**B**) depict the means ± SEM of fibril width (wt, n = 20; G33A, n = 17) and length (wt, n = 20; G33A, n = 17). ***p < 0.001, ns = not significant, Student’s t-test. (**C**) TEM micrographs showed that neither Aβ42 wt nor Aβ42 G33A formed mature fibrils when co-incubated with the AIP for 24 hours. (**D**) TEM analyses of Aβ42 wt peptides aggregated for 4 and 12 hours before addition of AIP and subsequent mixture was incubated for 24 hours. AIP was capable to block fibril formation of Aβ42 wt only when low-n oligomers had formed after 4 hours pre-incubation. AIP did not abolish fibril formation when higher aggregates or protofibrils were present (12 hours pre-incubation). (**C,D**) Scale bar = 100 nm. SEC analyses of aggregated Aβ42 peptides within 8 hours after freshly dissolving. (**E**) Aβ42 wt peptides mainly existed as tetra-/hexamers whose amount slightly decreased over time whereas the quantity of high-n oligomers (16–20mers) slightly increased. (**F**) Aβ42 G33A is presented by a higher amount of high-n oligomers, tremendously increasing over time. (**G**) Freshly dissolved Aβ42 wt peptide (0 h), after 4 and 8 hours of co-incubation with the AIP showed a constant amount of tetra-/hexamers and only a slight increase of high-n oligomers. (**H**) Co-incubation of Aβ42 G33A with the AIP abolished the shift from low aggregates to high-n oligomers over time. (**I**) AIP itself eluted from the column at 20 ml (for comparison see Fig. 1E,F).

To investigate the dynamics of Aβ42 aggregation, the effect of AIP on fibril formation was first examined by transmission electron microscopy (TEM). After 24 hours incubation at room temperature, Aβ42 wild-type (wt) peptides revealed well-structured, mature fibrils that were 18 nm wide and 300 nm long (Fig. 1A,B). In contrast, co-incubating Aβ42 wt peptides with AIP yielded protofibrillar-only structures and inhibited mature fibril formation (Fig. 1C). To further assess the targeting of Aβ42 aggregates by AIP, we incubated Aβ42 wt peptides for 4 or 12 hours prior to adding AIP, and then analyzed the aggregates for both time points after a total incubation period of 24 hours each. For the 4 hours/20 hours treatment, TEM analysis revealed oligomeric and protofibrillar structures without any Aβ42 wt mature fibrils. Interestingly, the formation of mature fibrils was not abolished in the 12 hours/12 hours treatment (Fig. 1D) suggesting that AIP prevents Aβ42 wt from undergoing structural transitions to a more compact form (i.e. characteristic of pre-fibrillar structures).

As an additional TEM control, we used the Aβ42 G33A substitution peptide that is known to form oligomeric beta-pleated sheet complexes more readily than Aβ42 wt peptide^16^ and stops aggregation at premature fibril stages due to its more hydrophobic surface. The Aβ42 G33A peptides did not yield mature fibrils after 24 hours, (Fig. 1A), as expected, but we did observe protofibrillar structures that were smaller compared to Aβ42 wt (average length of 100 nm versus 300 nm, respectively). In contrast, we did not observe any significant differences in protofibril width between Aβ42 G33A and Aβ42 wt (Fig. 1B).

Since TEM is limited in its deconvolution and magnification^28^, we also performed high-resolution atomic force microscopy (AFM) to further examine the influence of AIP on pre-fibrillar assemblies (i.e. protofibrils and oligomers). In the presence of AIP, the AFM analyses revealed globular particles and higher oligomeric structures with an average length of 100 nm for both Aβ42 and Aβ42 G33A peptides (Fig. S1). Validating our TEM data, this result implies that AIP might also affect oligomer formation at early stages of Aβ aggregation.

In order to acquire more detailed information at time points before fibrils are first observed, we performed size-exclusion chromatography (SEC) using experimental conditions to match our corresponding microscopic analyses. When freshly dissolved Aβ42 wt peptides were incubated for 0, 4, or 8 hours, slow oligomerization dynamics consistently yielded superimposable tetra-/hexamer oligomer peaks (Fig. 1E). The Aβ42 G33A substitution peptide exhibited faster kinetics (Fig. 1F), immediately forming high-n oligomers, which steadily increased over time, while the amount of tetra-/hexamers decreased. Thus, Aβ42 G33A exhibited a faster aggregation process over 8 hours compared to the wt peptide (Fig. 1F and E, respectively). When the identical Aβ42 wt experiments were repeated in the presence of AIP, consistent kinetics yielded superimposable tetra-/hexamer oligomer peaks with only a modest increase in high-n oligomers (compare Fig. 1E with 1G). Co-incubating Aβ42 G33A with AIP significantly reduced the formation of high-n oligomers over time while reducing the loss in low-n oligomers (compare Fig. 1F with 1H). Importantly, our findings establish that (i) the oligomer-stabilizing effect of AIP is stronger for the more aggregation-prone Aβ42 G33A peptide^16^ compared to Aβ42 wt, and (ii) AIP has a quantitative impact on oligomers in that it can attenuate the conversion of low-n tetra-/hexamers into high-n oligomers. Moreover, AIP appears to interact with tetra-/hexameric Aβ complexes with a 1:1 stoichiometry since we did not observe any other novel peaks eluting at different times. As an additional control, we confirmed that AIP elutes from the column in the salt fraction (Fig. 1I).

### AIP inhibition of Aβ42-induced neurotoxicity

**Figure 2:**
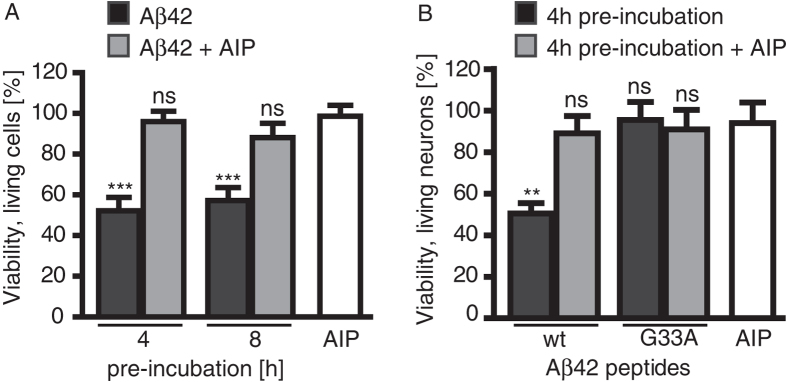
Cell viability of SH-SH5Y cells and primary hippocampal neurons after treatment with Aβ42 peptides. (**A**) SH-SY5Y cells were treated with 4- or 8-hour pre-incubated Aβ42 wt peptides (2.5 μM), in the presence and absence of AIP. Shown are graphs depicting the mean ± SEM viability as a % normalized to vehicle-treatment. ***p < 0.001, One-way ANOVA and Dunnett’s post-hoc test (AIP treatment was used as control). Number of independent experiments in triplicate: Aβ42 4 h, n = 4; Aβ42 + AIP 4 h, n = 7; Aβ42 8 h, n = 6; Aβ42 + AIP 8 h, n = 8. (**B**) Hippocampal primary neurons were treated with 4-hour preincubated Aβ42 wt or Aβ42 G33A peptides, in the presence and absence of AIP. The viability (%) is shown as the mean ± SEM, normalized to vehicle-treated cells. **p < 0.01, One-way ANOVA and Dunnett’s post-hoc test (AIP treatment as control). Number of independent experiments in triplicate: Aβ42 wt n = 11, Aβ42 wt + AIP n = 5, Aβ42 G33A n = 9, Aβ42 G33A + AIP n = 6, AIP n = 6.

Since AIP was able to interact with low-n oligomers and attenuate mature fibril formation in the experiments above, the effect of AIP on neurotoxicity was then examined using MTT assays in SH-SY5Y cells. In the absence of AIP, freshly dissolved Aβ42 wt peptides (pre-incubated for 4 or 8 hours) were able to reduce the number of living cells by approximately 40% (Fig. 2A); under similar assay conditions, the presence of AIP was able to neutralize the Aβ42 wt-induced toxic effects. Likewise in MultiTox assays (Fig. 2B) where primary hippocampal neurons were pre-incubated with Aβ42 wt in the absence and presence of AIP for 4 hours, AIP almost completely attenuated the Aβ42-mediated loss of living cells. As a control, we also tested the non-toxic Aβ42 G33A peptide^16^, which behaved similarly to Aβ42 wt with AIP.

**Figure 3:**
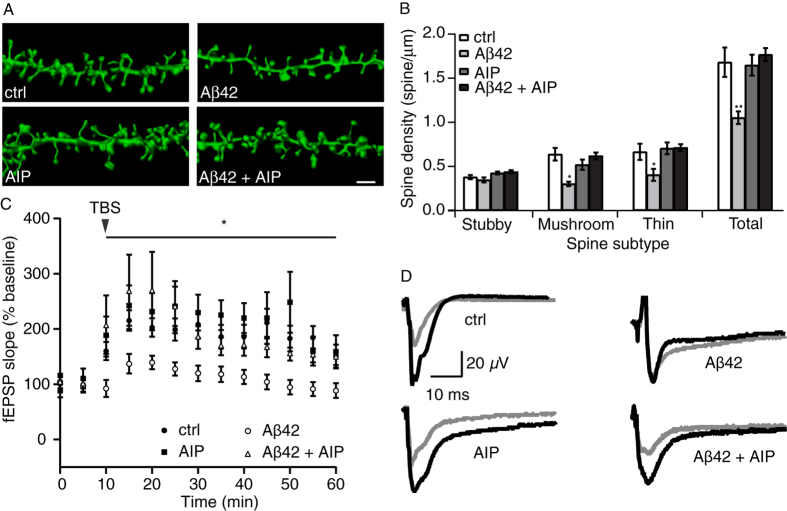
Quantification of dendritic spines and LTP recordings of Schaffer collateral CA1 region following Aβ42 and Aβ42 with AIP treatments. (**A**) Representative 3D reconstructions of dendritic segments from organotypic hippocampal sister slice cultures that were treated either with Aβ42 (1 μM), AIP or Aβ42 and AIP-containing media. Scale bar = 2 μm. (**B**) There is a significant decrease in the densities for spine subtypes following Aβ42 treatment compared to control. Treatment with Aβ42 in conjunction with AIP did not affect spine density. Total dendritic segment lengths (mean ± SEM): Control, n = of 672 μm from 12 cells in 4 cultures; Aβ42, n = 795 μm from 14 cells in 4 cultures; AIP, n = 884 μm from 16 cells in 4 cultures; Aβ42 + AIP, n = 970 μm of dendrite from 18 cells in 4 cultures. *p < 0.05, **p < 0.01 one way ANOVA with Tukey’s multiple comparison test was performed for each spine subtype (* refers to Aβ42 vs. Aβ42 + AIP). (C) AIP treatment in the presence of oligomeric Aβ42 species prevented the decrease in LTP level following TBS. Shown are quantifications of fEPSPs from control, Aβ42-, AIP-, and AIP + Aβ42-treated slice cultures. Following 24 hours of Aβ42 treatment (1 μM), there was a marked decrease in LTP response. This deficit was prevented by AIP (AIP + Aβ42). AIP alone did not have an effect on LTP. Control, n = 9 slices; Aβ42, n = 6 slices; AIP, n = 6 slices; AIP + Aβ42, n = 6 slices; One-way ANOVA with Tukey post-hoc comparison was used on the last time-point (*p < 0.05). fEPSP stimulus was delivered and recorded every 10 s; data points were averaged into five minute intervals for display and statistical analysis purposes. Averaged sample traces of fEPSPs obtained from control, Aβ42-, AIP-, and AIP + Aβ42-treated slice cultures are shown in (**D**). There was a marked decrease in the level of potentiation achieved following TBS stimulation. Black, baseline; grey, 50 minutes after TBS.

Given that soluble Aβ species can lead to dendritic spine loss^29^ and reduce spine plasticity at dendrites^17^, we examined the effect of AIP on the excitatory synapse stability of dendritic spines in the presence of Aβ42. Treating organotypic hippocampal slice cultures with Aβ42 for 24 hours led to a significant (p < 0.05) reduction in the number of CA1 pyramidal cell dendritic spines compared to control slices (Fig. 3A,B; mean ± SEM of spine/μm are as follows: control, 1.68 ± 0.17 spine/μm; Aβ42, 1.05 ± 0.07 spine/μm). Co-incubation with AIP, however, was able to prevent the Aβ42-mediated CA1 spine density losses (AIP, 1.65 ± 0.12 spine/μm vs. Aβ42 + AIP, 1.71 ± 0.07 spine/μm). Next, we investigated whether the significant decrease in spine density was subtype-dependent (i.e. thin, stubby, mushroom) in order to assess the number of glutamatergic α-amino-3-hydroxy-5-methyl-4-isoxazolepropionic (AMPA)-type receptors and synapse strength^30^, i.e. larger spine heads favor the presence of more AMPA receptors and stronger synapses^31^. Importantly, mushroom and thin spine subtypes were affected by Aβ42 treatment after 24 hours.

As LTP can be accompanied by structural modifications of dendritic spines, we next examined the effect of AIP on theta-burst stimulation (TBS)-induced LTP in the presence of Aβ42 in the CA1 region of mouse hippocampal slices. In keeping with previous findings, Aβ42 significantly inhibited LTP in the CA1 region of mouse hippocampal slices (Fig. 3C; p < 0.05), as compared to control (50 minutes post-TBS (mean ± SEM): control, 151 ± 21%; Aβ42, 88 ± 13%). Under similar conditions, LTP was not decreased when Aβ42 was co-incubated with AIP (50 minutes post TBS: AIP, 159 ± 30%; Aβ42 + AIP, 148 ± 14%). Figure 3D depicts averaged representative traces of field excitatory postsynaptic potentials (fEPSPs).

### AIP affects the *D. melanogaster* rough-eye phenotype and electrophysiology

**Figure 4:**
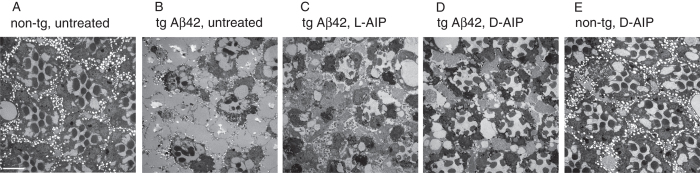
Ultrastructural analyses of retinas from Aβ42 wt-expressing *D. melanogaster* treated with AIP. Shown are representative electromicrographs of the ultrastructure of fly retinas in cross-section. Eyes of non-tg control flies showed a highly ordered structure of rhabdomeres and ommatidia (**A**). In contrast, flies expressing Aβ42 wt (**B**) have a rough-eye phenotype, with pronounced malformed ommatidia and rhabdomeres. Aβ42 wt tg flies raised on 5 mM L-AIP showed an improvement of rough-eye phenotype (**C**), but those raised on D-AIP (**D**) showed a far greater retention of ommatidia and rhabdomere structures, as compared to (**B**). (**E**) Treatment of non-tg control flies with D-AIP (5 mM) did not affect eye morphology. Scale bar = 5 μm.

To assess the effect of AIP *in vivo*, we utilized our previously developed *Drosophila melanogaster* model where fly strains expressing and secreting Aβ42 in the eye invokes an abnormal “rough-eye” phenotype^16^,^32^. The extent of cell death can be determined by visual inspection of the eye morphology^33^ and the severity of the Aβ42-induced toxicity can be estimated by the ratio of photoreceptors (rhabdomeres to ommatidia)^16^,^32^. TEM analysis of eye cross-sections from 5-day old non-transgenic flies revealed intact ommatidia with seven characteristic photoreceptor cells present (Fig. 4A). In contrast, TEM images from the Aβ42-transgenic flies exhibited severe distortions in eye morphology (Fig. 4B); specifically, the “rough-eye” phenotype was characterized by significantly altered ommatidia (smaller and less expressed), where the photoreceptors are less expressed and rhabdomeres appear shrunken.

To test the effect of AIP, we carried out food supplementation studies, where Aβ42-transgenic fly larvae were raised on food containing 5 mM AIP, which is a concentration that is in accordance to most studies examining the effects of drugs on flies^34^. Also, since living organisms typically catabolize L-amino acid-containing proteins, for these *in vivo* studies, we tested AIPs comprised of either L- (L-AIP) or D-amino acids (D-AIP). We found that AIP-treatment led to an attenuation of Aβ42-induced toxicity but not a complete rescue. For the flies that consumed AIP composed of L-amino acids (L-AIP; Fig. 4C), there still was diminished expression of photoreceptors in all ommatidia, vacuoles could be found, and shrunken rhabdomeres. For D-AIP-raised transgenic flies (Fig. 4D), eye morphology dramatically improved, including ommatidia with up to seven characteristic rhabdomeres, however, vacuoles could still be detected. As a control, the treatment of non-transgenic flies with D-AIP alone had no adverse effects on eye morphology (Fig. 4E). Since the D-AIP was significantly more effective at ameliorating the toxic phenotype, it can be regarded as a peptidomimetic—i.e. possessing similar selectivity and potency as the native L-AIP “parent”, as cross-validated in our *in vitro* systems (Fig. S2). The peptidomimetic was likely more effective in our Aβ42-transgenic fly model since it has been reported that the D-amino acids are more protease-resistant (i.e. increased stability of AIP) as compared to their L-amino acid counterparts^35^,^36^.

**Figure 5:**
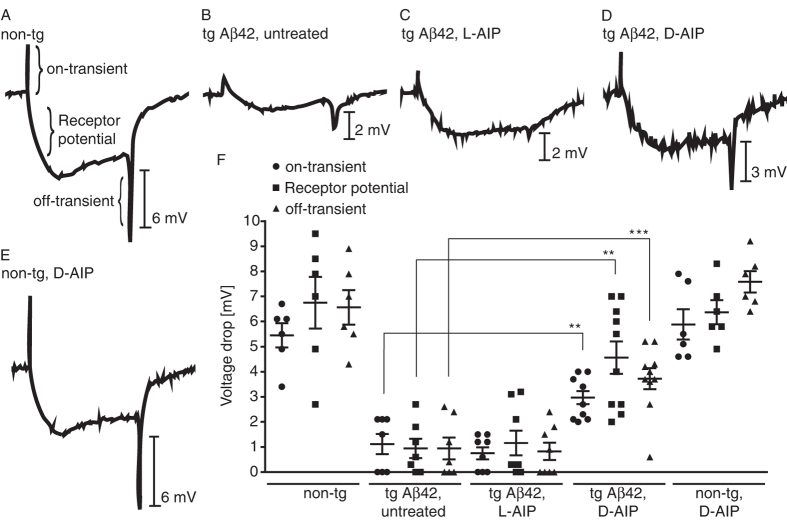
ERG traces in *D. melanogaster* expressing Aβ42 wt in the presence and absence of AIP. Shown are representative ERG traces from non-tg (**A**) or tg Aβ42-expressing flies in the absence (**A,B**) and presence of L-AIP (**C**), or D-AIP (**D,E**). (**A**) Untreated non-tg flies showed characteristic on- and off-transients, and receptor potentials. (**B**) Untreated tg Aβ42 flies revealed reduced on- and off-transients, and diminished receptor potentials. (**C**) Tg Aβ42 flies treated with 5 mM L-AIP exhibited almost no on- and off-transients, and no receptor potential. (**D**) Tg Aβ42 flies treated with 5 mM D-AIP demonstrated a significant response to light compared to untreated tg Aβ42 flies. (**E**) Non-tg flies treated with D-AIP showed no effect compared to untreated non-tg flies (**A**). Quantification of on- and off-transients, and receptor potential from each group are shown in (**F**). Data are represented as the mean ± SD of n = 6–10. **p < 0.01, ***p < 0.001, One way ANOVA with Tukey’s multiple comparison test.

To determine if the protective effect of D-AIP at the structural level (i.e. retinal morphology) translated to improved eye function in the treated Aβ42-transgenic flies, we then recorded electroretinograms (ERGs). All photoreceptor cells respond to a simple light pulse with a short delay, followed by a sustained depolarization (so-called “receptor potential”) that lasts as long as the stimulus^37^. The majority of the response is from photoreceptors R1-6 and their post-synaptic targets. Rhodopsin1 (Rh1) in R1-6 responds broadly to light with a peak in the blue range, R8 rhodopsin overlaps well with the Rh1 spectral sensitivity, and UV sensitive photoreceptors (R7) fire least robust to this stimulus^38^. Recorded with an electrode in the distal retina and a reference electrode elsewhere in the body, the ERGs measured as a change in the voltage drop across the basement membrane. Our non-transgenic flies (Fig. 5A) generated typical ERG profiles: on-transient signal (mean ± SD: 5.45 ± 1.18 mV), followed by a receptor potential (6.75 ± 2.52 mV), and finally the negative off-transient signal (6.57 ± 1.69 mV). Aβ42-expressing flies (i.e. transgenic; Fig. 5B) exhibited a significantly reduced response to light, as indicated by the missing on- and off-transient signals and receptor potential: 1.11 ± 1.06 mV, 0.94 ± 1.15 mV, and 0.94 ± 1.02 mV, respectively. Aβ42 transgenic flies treated with L-AIP (Fig. 5C) generated ERG profiles similar to the untreated Aβ42 flies (on-transient, 0.75 ± 0.68 mV; receptor potential, 1.16 ± 1.40 mV; off-transient, 0.83 ± 0.97 mV). Remarkably, treatment with D-AIP (Fig. 5D) significantly increased their response to light, as indicated by the enhanced on- and off-transient signals, and receptor potential (2.97 ± 0.81 mV, 3.72 ± 1.33 mV, and 4.56 ± 2.03 mV, respectively). Non-transgenic flies treated with D-AIP did not exhibit altered ERGs (Fig. 5E; on-transient, 5.88 ± 1.49 mV; receptor potential, 6.37 ± 1.18 mV; off-transient, 7.58 ± 1.04 mV), as compared to our untreated, non-transgenic flies (Fig. 5A). Figure 5F displays the quantifications of the on- and off-transient signals, as well as the receptor potential for each sample group.

### Analysis of AIP interaction with Aβ

**Figure 6:**
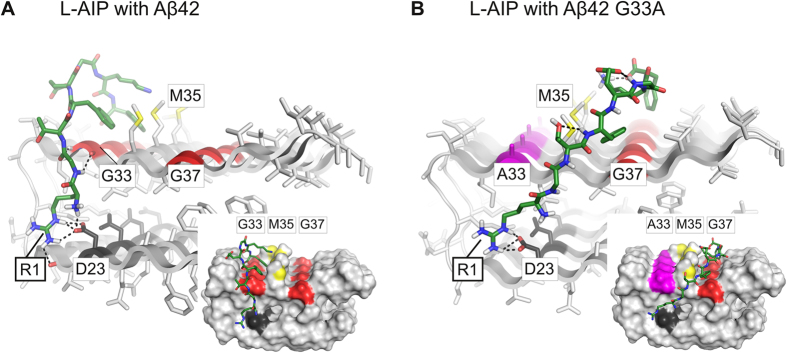
L-AIP docked to Aβ42 and to Aβ42 G33A. (**A**) Representative L-AIP Aβ42 complex obtained from flexible docking in stick (full view) or surface representation (inset). (**B**) Representative L-AIP Aβ42 G33A complex obtained from flexible docking in stick (full view) or surface representation (inset). In all figures, L-AIP is shown in green sticks with nitrogen colored in blue, oxygen in red, and hydrogens of polar side chains in white. Residue labels are marked by thick boundary lines. Hydrogen bonds are depicted as black dashed lines. Aβ42 and Aβ42 G33A are shown as gray cartoons and sticks, with G33 and G37 colored in red, Ala33 in magenta, Asp23 in black, and sulfur atoms of Met35 in yellow. Residue labels are marked by thin boundary lines.

Based upon our biophysical AIP data (TEM, AFM, SEC) and the Aβ42 structure provided by Luhrs and colleagues^39^, we generated molecular docking models for both L-AIP (Fig. 6, Fig. S3, S4) and D-AIP (Fig. S5), and its interaction with Aβ42 tetramers.

To predict how L-AIP might interact with Aβ42 tetramers, 6 best-fit poses were generated (see Methods). A strong interaction was found between AIP-Arg1 and Asp23 of the terminal sheet. Due to a lateral shift within the Aβ 42 tetramer, Asp23 of the terminal sheet is unpaired, while within the oligomer it forms a salt bridge with Lys28 of the neighboring sheet promoting fibril growth (Fig. 6A, S3A). In our model, L-AIP-Arg1 replaces Lys28 in that interaction, implying an interference with the Asp23-Lys28 salt bridge formation. The predominant configuration of L-AIP binding is with its C-terminal portion to the groove formed at Gly33 (Fig. 6A, see Fig. S4A–E). L-AIP-Arg1 extends down to Asp23, forming a strong hydrogen bond. Additional interactions are formed between Phe4 or 8 and Gly33 plus Met35 (Fig. 6A; S4A–E). In a second binding mode, which is less frequently found, L-AIP binds to the Gly37 groove, still forming the Arg1-Asp23 hydrogen bond (Fig. S3A; S4F).

Finally, docking of D-AIP to Aβ42 (Fig. S5A–G) yielded similar binding modes and specific interactions including the Arg1-Asp23 bond, as compared with the L-AIP (Fig. 6A) (Fig. S5A–C, and G). Due to the isomeric structure, however, we observe a slightly improved packing of D-AIP into each of the Gly grooves, forming similar interactions with Aβ42 (Fig. S5D–F).

In our second analysis, we performed flexible docking of L-AIP to the substitution peptide Aβ42 G33A to find a rationale for its faster aggregation and unique behavior compared to L-AIP (see Fig. 1E–H). The introduction of a methyl group to residue 33 changes the L-AIP binding to the Gly37 groove. Different interactions, including poses with reversed N- to C-terminal AIP topology (poses 1 and 2) are observed (Fig. S3B, S4G, H). As indicated in the third best-fit pose, configurations forming the Arg1-Asp23 hydrogen bond are still possible, although they less likely occur as with Aβ42 (Fig. 6B, S4I).

The tetramer is the minimal unit required to meet the steric/spatial constraints imposed by the length of the peptidic molecule, which binds to the groove provided by Gly33 and simultaneously interacts with Asp23.

**Figure 7:**
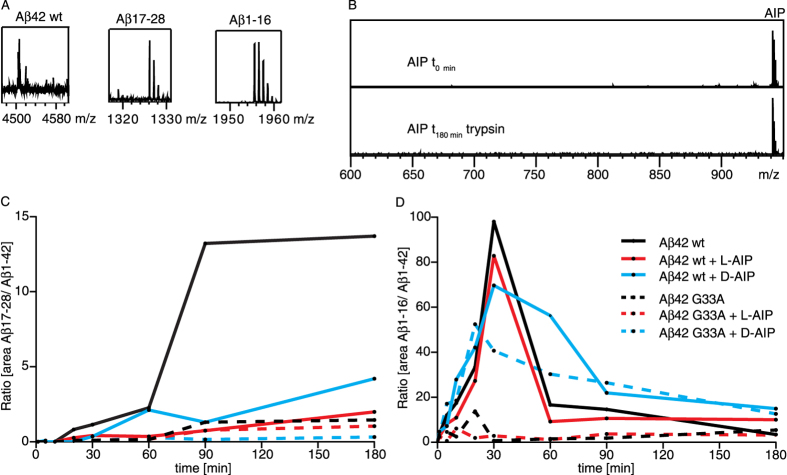
Co-incubation of Aβ42 with AIP affects accessibility of Lys28. (**A**) MALDI-MS spectra of tryptic digests yielded the following mass peaks (in Dalton (Da)): Aβ42 wt, 4512.3; Aβ17–28, 1325.7; Aβ1–16, 1954.9. (**B**) MALDI-MS spectra of a 3 hour tryptic digest of L- or D-AIP (941.5). Generation of Aβ17–28 (**C**) and Aβ1–16 (**D**) fragments by limited tryptic digestion of Aβ42 wt, Aβ42 G33A with or without L- or D-AIP, respectively. The values of the respective fragments were calculated as the ratio of ion peak areas of MALDI-MS spectra recorded at different time points. (**C**) For Aβ42 wt a rapid generation of high amounts of Aβ17–28 was observed, but hardly any cleavage of trypsin was seen at position Lys28 after co-incubation with L- or D-AIP. No difference was observed for Aβ42 G33A incubated with or without L- or D-AIP. (**D**) The ratios of the fragment Aβ1–16 generated from Aβ42 wt or Aβ42 G33A yielded no differences with or without L- or D-AIP, respectively.

To provide further evidence of the physical interaction between AIP and Aβ42 (as predicted by our SEC and molecular docking results), we performed limited proteolysis experiments. In brief, Aβ42 was incubated with trypsin and aliquots taken at different time points were analyzed by MALDI mass spectrometry (MS) to test for the presence of specific cleavage products. In the absence of AIP, the cleavage of Aβ42 generated Aβ1–16 and Aβ17–28 fragments and their corresponding C-terminal products (Fig. 7A), thus implying that this region is highly flexible and easily accessed by trypsin. To visualize the proteolytic time course, area under the curve of peaks from the acquired MALDI spectra were used to calculate the ratio of fragment Aβ17–28 to uncleaved Aβ42. Co-incubating Aβ42 with either L- or D-AIP led to a significantly decreased ratio of Aβ17–28 to Aβ1–42, most likely by protecting the Aβ42 peptide from trypsin by shielding the Lys28 cleavage site (Fig. 7C). Since AIP itself contains two basic residues (Arg in position 1 and Lys as the second last amino acid residue at the C-terminus), we also performed L- and D-AIP-only trypsin digestions (i.e. without Aβ42) to ensure that AIP was not degraded during the three hour time-course (Fig. 7B). Overall, our proteolysis data (i.e. masking of Lys28 in Aβ42 by AIP) is consistent with our previous Aβ42 G33A studies where the substitution of glycine 33 to alanine induced a more tightly packed Aβ42 conformation that masks Lys28^16^. Accordingly, co-incubating Aβ42 G33A with L- or D-AIP did not significantly change the ratio of Aβ17–28 to Aβ1–42 G33A (Fig. 7C). It is also important to note that AIP did not affect general trypsin activity, as evidenced by the ratio of fragment Aβ1–16 to uncleaved Aβ42 which was unaltered in the absence or presence of AIP (Fig. 7D).

**Figure 8:**
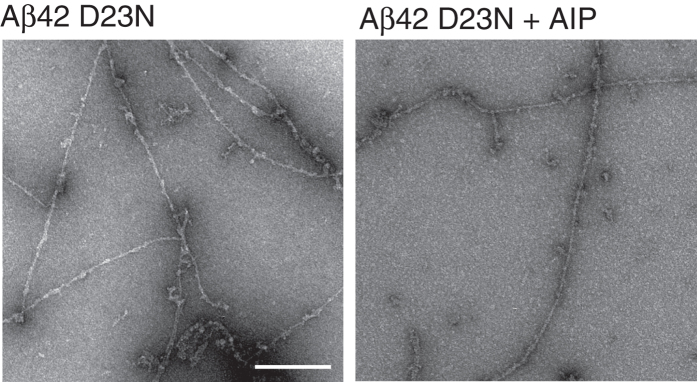
Aggregation studies of Aβ42 D23N peptides. TEM micrographs were acquired after 24 hours incubation. Aβ42 D23N aggregated to mature fibrils. AIP did not inhibit fibril formation of Aβ42 D23N. Scale bar = 200 nm.

Since the Asp23-Lys28 salt bridge appears to be important for AIP to inhibit Aβ42-mediated toxicity, we tested the Aβ42 D23N Iowa mutation peptide which is associated with progressive AD-like dementia, and has been found to be more neurotoxic and to aggregate more rapidly than the wt peptide^40^. In the Asp-to-Asn mutant, the wild-type Asp23-Lys28 salt bridge cannot form, thus allowing the sequence to transform either in parallel or antiparallel β-sheets which alters its folding nucleation^41^. Indeed, elimination of the salt bridge in the Asp23 to Asn substitution peptide abolished the ability of AIP to inhibit mature fibril formation as revealed by EM (Fig. 8). Notably, this is fully consistent with our best-fit molecular docking poses implying that AIP-Arg1 and its protonated N-terminus make H-bonds to Asp23, interfering with the Asp23-Lys28 salt bridge.

## Discussion

The current Aβ oligomer hypothesis implies that oligomeric Aβ species are toxic while amyloid plaques/fibrils are not inherently toxic but a consequence of aberrant Aβ aggregation^42^. Accordingly, to investigate molecular mechanisms of actions that can detoxify these neurotoxic oligomers is important and highly desirable. In this study, we have demonstrated *in vitro* and *in vivo* that a non-amidated 8-residue peptide (RGTFEGKF) can function as a selective and potent Aβ-oligomer Interacting Peptide (AIP). AIP not only suppresses aggregation of Aβ oligomers into proto-fibrillar structures, but also neutralizes toxic Aβ oligomers. AIP directly influences the self-aggregation and *in vivo* toxicity of Aβ without leading to an obvious disaggregation of amyloid oligomers from fibrils. Oligomers were stable in the presence of excess AIP. In this regard, AIP might act as a chaperone, not causing an apparent dissociation of Aβ complexes into monomers, but competing with the addition of further monomers or oligomers to effectively prevent the growth of Aβ oligomers into larger assemblies. Compared to previously reported anti-amyloid oligomer strategies, AIP is unique in that it is neither a dissociation catalyst nor a monomer competitor (e.g antibodies such as crenezumab or solanezumab^43^) nor an aggregation enhancer (e.g. D-amino peptides such as D3^44^).

Our biophysical data (EM, AFM, SEC) together with limited proteolysis/mass spectrometry results demonstrate that AIP directly interacts with Aβ. While the amidated predecessor of AIP was initially designed on the framework GxFxGxF to disrupt sheet-to-sheet packings of Aβ40 fibrils^26^,^27^, our non-amidated AIP can inhibit mature fibril formation, suppress large aggregate formation, and appears to specifically interact with Aβ tetramers. In contrast to other small aggregation inhibitors^45^,^46^,^47^, AIP shows that peptide-based compounds can arrest further growth of the aggregates – an important and beneficial mode of action. To not only bind pre-existing low-n oligomeric complexes (with varying degrees of toxicity and solubility), AIP also may target a multitude of toxic Aβ oligomeric species built from potentially toxic subunits of Aβ tetramers^15^,^16^,^48^. As demonstrated by our EM and AFM data, co-incubating AIP with Aβ inhibits the formation of mature fibrils (ultimately the same outcome as the substitution variant Aβ42 G33A alone, as described previously^16^).

The Aβ42 G33A tetramer is naturally non-toxic^16^,^49^,^50^ and has a strong tendency to assemble into high-n oligomers due to its extended hydrophobic surface patch. The substitution at position 33 may cause an altered seed structure of Aβ42^51^ and substitution variants with either an alanine or isoleucine at position G33 of the Aβ sequence showed a lack of Aβ42-induced neurotoxicity, but an increased aggregation of freshly dissolved peptides due to structural differences^16^,^49^. Positions Ile31, Gly33 and Met35 have an overall higher solvent-excluded surface value and especially shielding of Gly33 leads to an enlarged solvent-excluded surface and promotes aggregation^16^. In the present study, we show that Gly33 not only impacts the aggregation of freshly dissolved Aβ42 peptides, it provokes the formation of high-n oligomers over time as observed by our SEC studies. However, our EM and AFM data show that Aβ42 G33A does not form mature fibrils and, in this regard, its behavior is similar to AIP/Aβ42 co-incubation.

It has been shown that Aβ42 oligomers can disrupt LTP in slices and *in vivo*, and also have the potential to impair the memory of a complex learned behavior in rats^3^,^16^,^17^. Furthermore, learning models such as LTP have been shown to induce dendritic spine structural modifications^52^,^53^, and LTP induction has been demonstrated to promote spine enlargement and the growth of new spines^31^,^54^,^55^,^56^,^57^, spine branching^58^, and actin-dependent remodeling^59^,^60^. Notably, an increase in spine volume accompanied by insertion of AMPA receptors has been observed as well^31^. Since Aβ42 low-n oligomers promote the progressive loss of dendritic spines and glutamatergic synapses^17^,^18^,^61^, our present study (spine density, LTP, and toxicity data combined) clearly demonstrates that AIP can neutralize Aβ42-induced neurotoxic effects. Mechanistically, it is likely that AIP masked the receptor recognition site of Aβ42 oligomers, e.g., the cellular form of the prion protein, which has been suggested as a receptor for Aβ42 oligomers^62^. Consequently, Aβ42 “trapped” oligomers would not interact with the putative receptor, which may result in non-altered spine morphology and no decreased LTP, as demonstrated in our study.

The *Drosophila* “rough eye” model has become a well-established system to study toxic events as related to human cancers^63^ and neurodegeneration^64^. To assess whether the AIP has a protective effect against cell-derived Aβ42, we used our previous *Drosophila* model where the transgenic expression and secretion of Aβ42 in the eye generates Aβ aggregates^16^. Transgenic Aβ42 flies raised on food containing D-amino acid AIP exhibited a less severe rough-eye phenotype compared to (i) transgenic Aβ42 flies treated with L-amino acid AIP, or (ii) non-treated transgenic Aβ42 flies. In addition to its small molecular mass (<1 kDa), the added protease resistant nature of D-AIP most likely contributed to its superior bioavailability and efficacy^35^,^36^. Since D-AIP could attenuate Aβ42-induced toxicity at both structural (i.e. significantly improved eye morphology by EM) and functional levels (i.e. significantly ameliorated function of photoreceptors by ERG), our findings underscore the potential of the AIP as a new therapeutic strategy.

Our *in vitro* and *in vivo* results can be explained by the interaction of AIP with the two Aβ42 sites (Gly33 and Asp23) that are critical for fibril growth and modulating toxicity. The molecular docking poses indicate that both L- and D-AIP make multiple hydrogen bonds to free backbone amid nitrogens or oxygens at the oligomer edges with either polar side chains or the backbone, which further hinders Aβ42 β-sheet attachment. Similarly, others have identified tau residues 306–311 (VQIVYK) as a target template for the D-amino acid hexapeptide TLKIVW that can prevent self-interaction of the VQIVYK sequence via a steric hindrance^65^.

The best-fit docking poses for AIP revealed that AIP-Arg1 and its protonated N-terminus make H-bonds to Aβ-Asp23, which interferes with the Asp23-Lys28 salt bridge which is important for Aβ42 fibril growth. A peptide carrying the familial AD (FAD) mutation Aβ42 D23N (Iowa), unable to form the salt bridge by the substitution of Asp23 to Asn23^41^, failed to show the inhibitory effect of AIP on aggregation. This finding underscores the importance of the intramolecular contact between positions Asp23 and Lys28 in the Aβ sequence which alters aggregation and modulates toxicity since this FAD mutation has a 2- to 3-fold higher neurotoxicity compared to Aβ42 wt^40^. Thus, our data indicate that AIP contacts Aβ42 peptide through the Gly33- or the Gly37-groove and that the altered three-dimensional shape between L- and D-forms retained the biological activity of AIP, i.e., by interfering with the salt bridge and inhibiting Aβ-induced neurotoxicity.

Numerous molecules have been identified which possess the ability to prevent amyloid fibril formation, both *in vivo* and *in vitro*. These molecules range from large proteins such as molecular chaperones^19^ to small molecules such as flavonoids^20^ to polyphenols^21^. So far, the most successful structure-based approach to prevent fibril formation has been to stabilize the native tetrameric structure of transthyretin (TTR)^66^ in TTR amyloidogenesis. Tafamidis meglumine, a small molecule which binds selectively to two normally unoccupied thyroxine-binding sites of the tetramer and kinetically stabilizes TTR through hydrophobic and electrostatic interactions^67^, similar to AIP-Aβ interactions. Although Aβ lacks a fully ordered native structure^68^, this effect proves the concept that stabilization of a native stable conformation can have beneficial outcomes in protein misfolding diseases, i.e., preparing AIP-Aβ complexes for removal and efficient clearance. While our present study establishes a clear biological effect of AIP on Aβ42-mediated toxicity, further investigation is needed to determine the exact interaction model between AIP-like inhibitors and amyloidogenic peptides for rational therapeutic design.

## Methods

### Peptides

Synthetic Aβ peptides and AIP were purchased from Peptide Specialty Laboratories, Germany, and ChemImpex INT’L INC, USA, respectively. Aβ was monomerized and solubilized as described^49^. Briefly, monomerized peptides were dissolved to 1 mg/ml in deionized water supplemented with ammonia to a final concentration of 0.13% (measured pH 9.8). The AIP was applied at an Aβ42-to-AIP molar ratio of 1:20.

### Transmission Electron Microscopy (TEM)

Aβ peptides were dissolved to 40 μM and incubated for 24 h at room temperature. Aliquots (10 μl) of matured peptide solutions were applied to glow-discharged carbon-coated copper grids, or to formvar-coated nickel grids, respectively, and negatively stained with 2% aqueous uranyl acetate as described^69^. Micrographs were taken using a Philips CM100 electron microscope at 100 kV and a Fastscan CCD camera (Tietz Video and Image Processing Systems GmbH, Gauting, Germany).

### Atomic Force Microscopy (AFM)

AFM is an appropriate method for the characterization of pre-fibrillar protein assemblies. Here, 5 μl of a 40 μM Aβ peptide solution were applied on a 0.5 mm^2^ freshly cleaved sheet of mica for 10–30 seconds and then removed by fast spinning off. AFM images were recorded using a MultiMode scanning probe microscope (either NanoScope IIIa, Digital Instruments Inc., or NanoScope IV, Veeco Instruments Inc., Santa Barbara, California, USA), equipped with a 10 μm scanner (E-scanner). Height and phase images were recorded in tapping mode with scan rates of 2–4 lines per second and a resolution of 512 × 512 pixels. Olympus etched silicon cantilevers were used with a typical resonance frequency in the range of 60–80 kHz and a spring constant of 2 N/m. All samples were investigated on dry substrates of mica (PLANO W. Plannet GmbH, Wetzlar, Germany) at room temperature open to air.

### Size exclusion chromatography (SEC)

SEC was performed as described^16^. Briefly, synthetic Aβ peptides were dissolved and mixed with or without AIP prior to incubation for 0, 4, or and 8 hours at room temperature. Oligomers were separated by a Superose 12 10/300 GL column (GE Healthcare, Germany) with PBS as running buffer at a flow rate of 0.5 ml/min.

### Limited proteolysis

Proteolysis was performed as described^16^ with minor modifications. Briefly, Aβ42 peptides were dissolved as described above and diluted in 25 mM ammonium bicarbonate, pH 7.5 to a concentration of 20 μg/ml. Aβ peptides were pre-incubated for 4 hours at room temperature without and in conjunction with the AIP, respectively. The tryptic digest was performed at 37 °C using an enzyme–substrate ratio of 1:500 trypsin (Roche) for up to 3 hours. Fragments were identified by mass spectrometry (UltrafleXtreme MALDI-TOF/TOF Bruker Daltonics, Germany) in reflector-positive mode using CCA matrix.

### Molecular Modeling

A tetrameric Aβ42 fibril core structure was obtained from the pentameric NMR structure 2BEG^39^ by removing one lateral beta sheet in Pymol. Aβ42 G33A tetramer was generated by point mutating the tetramer Aβ42 structure at all Gly33 to Ala using Schrödingers maestro 2014–2 suite (Schrödinger Release 2014–2: Maestro, version 9.8, Schrödinger, LLC, New York, NY, 2014). For docking, both structures were prepared with the Protein Preparation Wizard^70^.

A docking grid suitable for peptide docking was generated with its centre (10 Å) between the middle of two Met35, defining the center of all Gly/Ala33, Met35 and Gly37 residues by means of the receptor grid generator. This defines a binding site large enough for binding of the AIP ligand (with length <36 Å). The AIP was built as random coil in Pymol with charged Arginine, Glutamic acid and Lysine side chains and termini. Maestros build utility was used to generate its D-enantiomer (D-AIP). Both ligands were prepared with the LigPrep Wizard (LigPrep, version 3.0, Schrödinger, LLC, New York, NY, 2014) for the forcefield OPLS_2005^71^ including Epik protonation states^72^ and including the original state for neutral pH 7 ± 0.5 (covering physiological pH 7.4). Glide SP-peptide docking for flexible ligand sampling^73^ was performed using standard settings and specifically sampling nitrogen inversions, ring conformations (including input conformation), no bias sampling of torsions, and enhanced planarity of conjugated pi groups. The docking score was modified by Epik state penalties. The SP-peptide docking protocol yields around 119–200 poses per run with no more than 5–19 reasonable <-5 kcal/mol and 1–4 best docking scores <-7 kcal/mol.

### Toxicity on primary hippocampal neurons and neuroblastoma cells

Primary neurons were prepared as previously described^16^. Briefly, hippocampi of postnatal day 0 (P0)–P1 Wistar rat pups were dissociated and cultured in neurobasal A medium. After 10 days *in vitro*, Aβ42 (2.5 μM) peptides were pre-incubated (4 or 24 hours) in the presence and absence of the AIP, respectively. Neuronal cultures were treated with the pre-incubated peptides for 48 hours. Neuronal viability was detected using the MultiTox-Fluor Multiplex Cytotoxicity assay (Promega, Germany), performed according to manufacturer’s instructions.

To determine Aβ toxicity in SH-SHY5 cells, the MTT (3-(4,5-dimethylthiazol-2-yl)-2,5-diphenyl-tetrazolium bromide) assay was performed as described^49^. Aβ peptides were applied at a concentration of 2.5 μM for 12 hours.

### Organotypic hippocampal slice cultures, image acquisition and quantification of dendritic spines

Hippocampal slices (400 μm) from P6–8 transgenic mice expressing membrane-targeted MARCKS-eGFP under the Thy-1 promoter in a subpopulation of CA1 cells^74^ were prepared as previously described using the roller-tube method^75^. Slice cultures were maintained in the incubator with a roller-drum for three weeks before experimentation to allow for spine maturation^76^. Slices were incubated with or without 1 μM Aβ42 peptide and 20 μM AIP-containing media for 24 hours. Image acquisition and quantification was performed as described^76^. Briefly, following treatment, slice cultures were transferred to a temperature-controlled chamber (30 °C) mounted on an upright confocal microscope and continuously perfused with Tyrode solution. Secondary and tertiary dendritic branches from either apical or basal dendrites of CA1 pyramidal neurons were imaged.

All animal handling procedures were carried out in accordance with guidelines set by the Canadian Council on Animal Care and the National Institutes of Health in the USA. All procedures were approved by the Animal Resource Committee of the School of Medicine at McGill University, and are outlined in McGill University Animal Handling Protocol #5057.

### fEPSP recordings and Long Term Potentiation

fEPSPs were recorded in the CA1 *stratum oriens* at 30 °C as previously described^76^ with minor modifications. Briefly, a surgical cut was made between CA3 and CA1. Schaffer collateral axons were then stimulated with an insulated platinum–iridium bipolar electrode. The stimulus strength was between 40 μA and 200 μA, to achieve one-half to one-third of the maximum response, and stimulation was provided every 10 s. Data points were then pooled and averaged into five minute intervals for statistical analysis and graphical display in the figures. LTP was induced with a TBS paradigm consisting of bursts of five pulses at 100 Hz; the bursts were applied five times at intervals of 200 ms, and delivered five times every 10 s. Slices were treated with freshly-dissolved 1 μM Aβ42 peptide, 20 μM AIP, or 1 μM Aβ42 peptide and 20 μM AIP-containing media for 24 hours prior to recordings.

### Eye structure analysis of *D. melanogaster*

UAS-Aβ42 flies were generated as previously described^16^. Briefly, transgenic flies harboring UAS-Aβ42 were crossed with flies containing GMR-GAL4 to achieve eye-specific Aβ42 expression. Flies were bred on Jazz-Mix Drosophila fly food (Fisher Scientific, USA) containing 5 mM L-AIP or D-AIP, respectively. Five-day-old flies were collected and analyzed. Fly eyes were fixed in 2.5% glutaraldehyde, 2% formaldehyde in 100 mM PB buffer, pH 7.4, post-fixed in 1.5% osmium tetroxide. Samples were embedded in epon and polymerized at 60 °C for at least 48 hours. The sections were counterstained with aqueous uranyl acetate followed by lead citrate. Micrographs were taken using a Philips CM120 electron microscope at 80 kV and a 1 K CCD camera.

### Electroretinography

Flies were immobilized inside the end of a plastic pipette tip. The ERGs were recorded using borosilicate pipette microelectrode in contact with the eye, and a reference tungsten electrode inserted into the abdomen. The microelectrode was filled with artificial cerebral-spinal fluid containing: 115 mM NaCl, 5 mM KCl, 23 mM glucose, 26 mM sucrose, 4.2 mM HEPES, 2.5 mM CaCl_2_, and 1.3 mM MgCl_2_, pH 7.2. Signals were amplified and filtered (1 kHz) using an Axopatch 200B amplifier, sampled at 5 kHz using a Digidata 1440A, and recorded with Clampex 10.3 (Molecular Devices). Blue light from a LUXEON^®^Star LED with a dominant wavelength of 470 nm was delivered to the fly head via an optical fiber cable 5 cm from the fly head. Light stimulus was driven by a Thor labs LEDD1B controller with stimulus of 1000 ms. Representative traces were low bandpass filtered.

### Statistical Analyses

All results are expressed as means ± SEM (Standard Error of Mean). Statistical comparisons were made by either unpaired Student’s t-test, or by One-way ANOVA followed by Tukey-Kramer or Dunnett’s *post hoc* test to identify differences between treatments. All data presented are from a minimum of three independent experiments. P values < 0.05 were considered significant: *p < 0.05, **p < 0.01; ***p < 0.001.

## Additional Information

**How to cite this article**: Barucker, C. *et al.* Aβ42-oligomer Interacting Peptide (AIP) neutralizes toxic amyloid-β42 species and protects synaptic structure and function. *Sci. Rep.* **5**, 15410; doi: 10.1038/srep15410 (2015).

## Supplementary Material

Supplementary Information
