# Supplementary material for: Aβ42-oligomer Interacting Peptide (AIP) neutralizes toxic amyloid-β42 species and protects synaptic structure and function
Source: Sci Rep. 2015 Oct 29;5:15410. doi: 10.1038/srep15410 (PMC4625140; doi:10.1038/srep15410)

SUPPLEMENTAL INFORMATION

Aβ42-oligomer Interacting Peptide (AIP) neutralizes toxic amyloid-β42 species and protects synaptic structure and function

Christian Barucker[1,†], Heiko J. Bittner[2], Philip K.-Y. Chang[1], Scott Cameron[3,8], Mark A. Hancock[1], Filip Liebsch[1], Shireen Hossain[1], Anja Harmeier[4,9], Hunter Shaw[3], François M. Charron[1], Manuel Gensler[5], Paul Dembny[4,10], Wei Zhuang[5,11], Dietmar Schmitz[6], Jürgen P. Rabe[5], Yong Rao[3], Rudi Lurz[7], Peter W. Hildebrand[2], R. Anne McKinney[1] & Gerhard Multhaup[1,4]

[1]Department of Pharmacology and Therapeutics, Faculty of Medicine, McGill University, Montreal, QC, Canada.

[2]Institute of Medical Physics and Biophysics, Charite-Universitätsmedizin, Germany.

[3]Department of Neurology and Neurosurgery, McGill Centre for Research in Neuroscience.

[4]Institut für Chemie und Biochemie, Freie Universität Berlin, Germany.

[5]Department of Physics & IRIS Adlershof, Humboldt-Universität zu Berlin, Germany.

[6]Neurowissenschaftliches Forschungszentrum, Charite-Universitätsmedizin, German Center for Neurodegenerative Diseases (DZNE), Berlin, Germany.

[7]Max-Planck-Institute for Molecular Genetics, Germany.

[†]Deceased.

[8]Present address: Center for Neuroscience, University of California Davis, Davis, USA.

[9]Present address: F. Hoffmann-La Roche AG, Pharma Research & Early Development, Discovery and Translational Area Neuroscience Basel, Switzerland.

[10]Present address: Charite-Universitätsmedizin Berlin, Campus Mitte, Department of Neurology, Germany.

[11]Present address: BASF Polyurethane Specialties, Pudong of Shanghai, China.

Correspondence and requests for materials should be addressed to G.M. (email: gerhard.multhaup@mcgill.ca)

SUPPLEMENTAL FIGURE LEGENDS

Fig. S1: AFM micrographs showed no fibril formation of Aβ42 wt and Aβ42 G33A when co-incubated with the AIP. Both Aβ42 wt and Aβ42 G33A co-incubated with AIP were present as oligomeric and globular structures (scale bar = 100 nm). Without AIP (scale bar = 800 nm) Aβ42 formed mature fibrils after 24 hours incubation, while there was no difference between Aβ42 G33A incubated with or without AIP. Solely oligomeric and globular structures were detected for Aβ42 G33A after 24 hours incubation.

Fig. S2: Effects of D-AIP on Aβ42 wt aggregation. (A) TEM analyses showed that Aβ42 wt aggregation was inhibited by D-AIP. Scale bar = 100 nm. (B) SEC analyses of aggregation behavior of Aβ42 wt peptides co-incubated with AIP for 0, 4 or 8 hours. Aβ42 wt peptides were mainly present as tetra-/hexamers, which slightly increased in size after 8 hours of incubation. (C) SH-SY5Y cells were treated for 12 hours with 4- or 8-hour preincubated Aβ42 wt peptide, in the presence and absence of D-AIP, and the cell viability was determined using MTT assay. Co-incubation with AIP neutralized Aβ42 wt-induced neurotoxic effects, while AIP itself was non-toxic. The graph depicts the mean ± SEM of viability expressed as a %, normalized to vehicle-treated cells. *** $p<0.001$, One-way ANOVA followed by Dunnett's multiple comparison *post-hoc* test was performed (D-AIP treatment as control). Number of independent experiments: Aβ42 4 h, n = 7; Aβ42 + AIP 4 h, n = 9; Aβ42 8 h, n = 12; Aβ42 + AIP 8 h, n = 13; AIP, n = 3.

Fig. S3: Possible poses of L-AIP to Aβ42 and Aβ42 G33A docking. (A) L-AIP-Aβ42 complex obtained from flexible docking, top pose #6. L-AIP is in green sticks representation with nitrogen colored in blue, oxygen in red and hydrogens of polar side chains involved in H-bonds (dashed lines) in yellow. Gly33 and Gly37 are in red and marked with arrows, and Asp23 in black. Sulfur of Met35 is colored yellow. Visible N- and C-termini and interacting residue are shown in normal font for Aβ42 and in italics for L-AIP. In the inset, Aβ42 is

depicted in surface representation (in gray) and Gly33 and Gly37 in red, Asp23 in black and Met35-sulfur in yellow. (B) AIP-Aβ42 G33A complex obtained from flexible docking, top two poses. L-AIP is in green sticks representation with nitrogen colored in blue, oxygen in red and hydrogens of polar side chains involved in H-bonds (dashed lines) in yellow. Ala33 is in magenta and Gly37 in red and marked with arrows, and Asp23 in black. Sulfur of Met35 is colored yellow. Visible N- and C-termini and interacting residues are in normal font for Aβ42 G33A and in italics for L-AIP. In the inset Aβ42 G33A is depicted in surface representation (in gray) and Ala33 in magenta, Gly37 in red, Asp23 in black and Met35-sulfur in yellow.

Fig. S4: Preferred poses of L-AIP to Aβ42 and Aβ42 G33A docking. (A-F) L-AIP-Aβ42 complexes obtained from flexible docking, top six poses. L-AIP is in green sticks representation with nitrogen colored in blue, oxygen in red and hydrogens of polar side chains in white. Aβ42 is depicted in surface representation (in gray) and Gly33 and Gly37 in red, Asp23 in black and Met35 sulfur in yellow. N- and C-termini of Aβ42 are marked with N or C. (G-I) L-AIP-Aβ42 G33A complexes obtained from flexible docking, top three poses. L-AIP is in green sticks representation with nitrogen colored in blue, oxygen in red and hydrogens of polar side chains in white. Aβ42G33A is depicted in surface representation (in gray) and Ala33 in magenta, Gly37 in red, Asp23 in black and Met35 sulfur in yellow. N- and C-termini of Aβ42G33A are marked with N or C.

Fig. S5: Preferred poses of D-AIP to Aβ42 docking. (A-G) D-AIP-Aβ42 complexes obtained from flexible docking, top seven poses. D-AIP is in green sticks representation with nitrogen colored in blue, oxygen in red and hydrogens of polar side chains in white. Aβ42 is depicted in surface representation (in gray) and Gly33 and Gly37 in red, Asp23 in black and Met35-sulfur in yellow. N- and C-termini of Aβ42 are marked with N or C.

Figure S1

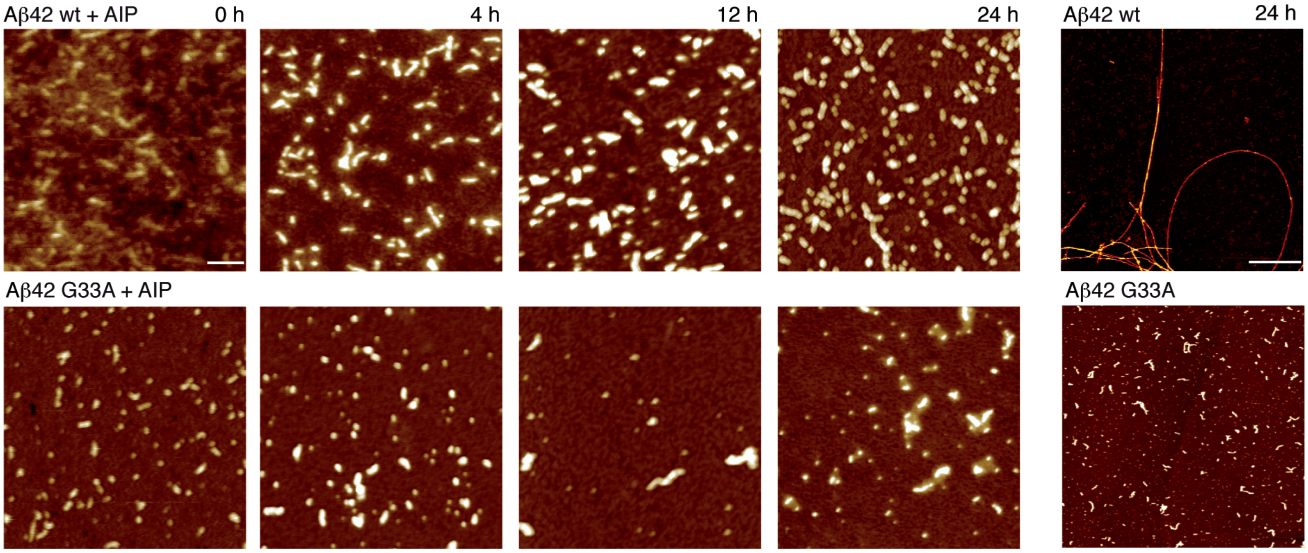

Suppl. Fig. 2

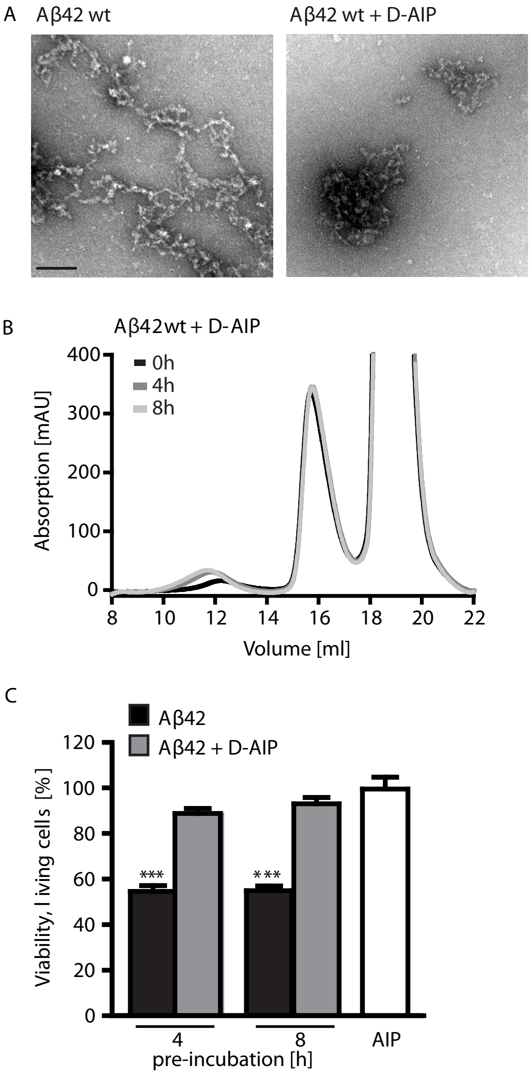
A
Aβ42 wt
Aβ42 wt + D-AIP
B
Aβ42wt + D-AIP
0h
4h
8h
Absorption [mAU]
400
300
200
100
0
8
10
12
14
16
18
20
22
Volume [ml]
C
Aβ42
Aβ42 + D-AIP
Viability, l iving cells [%]
120
100
80
60
40
20
0
***
***
4
8
AIP
pre-incubation [h]

Figure S3

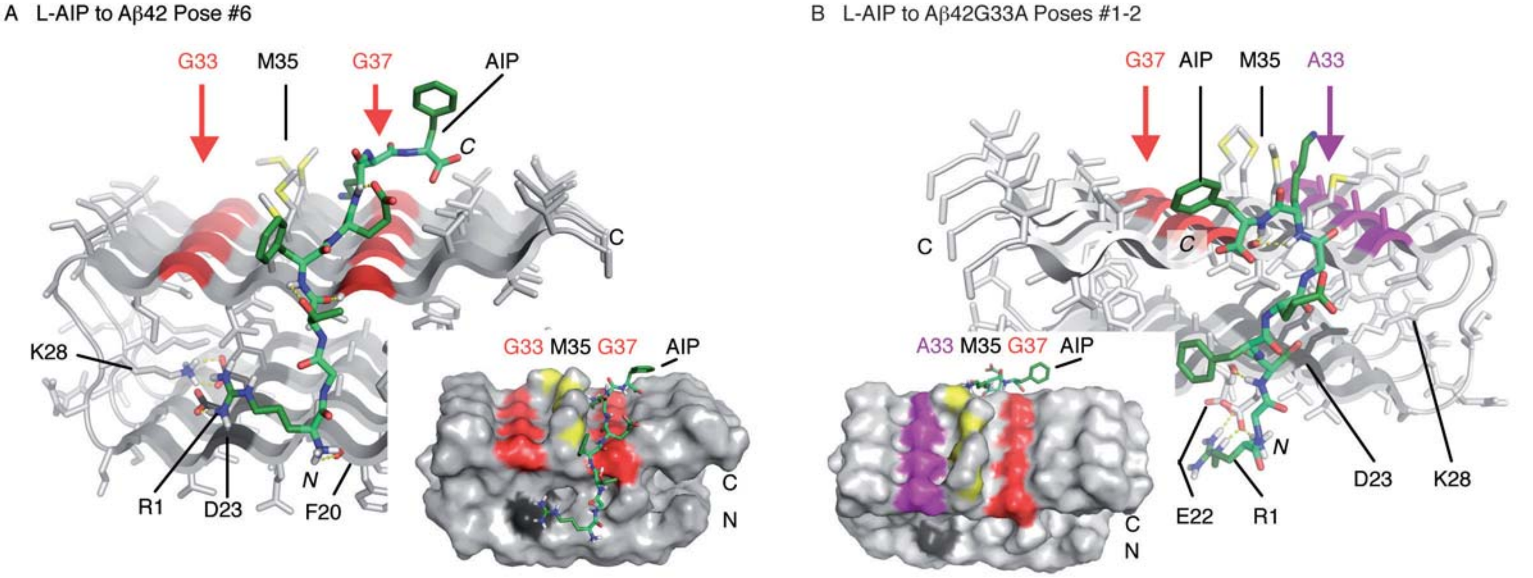

A L-AIP to Aβ42 Pose #6
G33
M35
G37
AIP
C
C
K28
R1
D23
N
F20
G33 M35 G37
AIP
C
N
B L-AIP to Aβ42G33A Poses #1-2
G37
AIP
M35
A33
C
C
A33 M35 G37
AIP
N
C
N
E22
R1
D23
K28

Figure S4

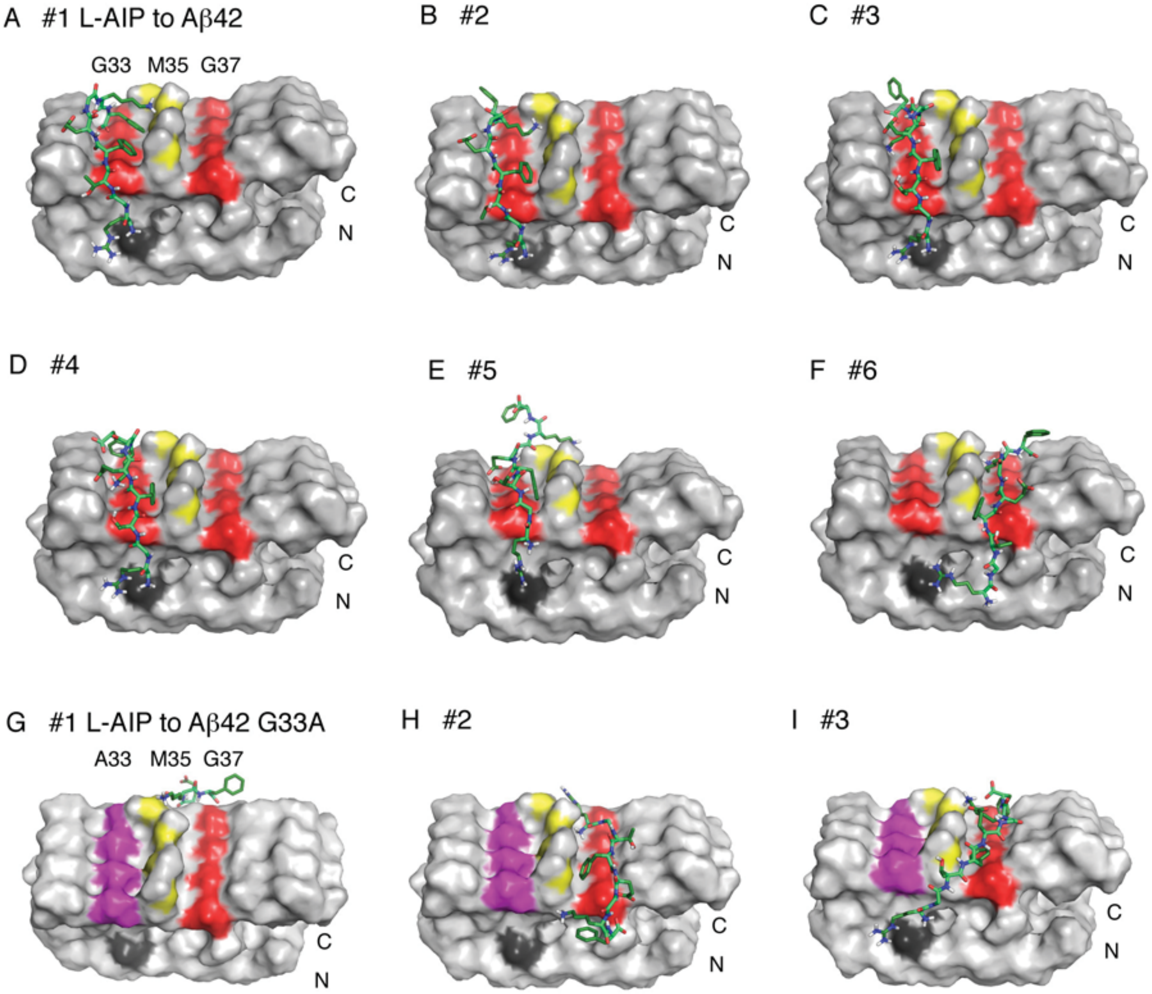
A #1 L-AIP to Aβ42
G33
M35
G37
C
N
B #2
C
N
C #3
C
N
D #4
C
N
E #5
C
N
F #6
C
N
G #1 L-AIP to Aβ42 G33A
A33
M35
G37
C
N
H #2
C
N
I #3
C
N

Figure S5

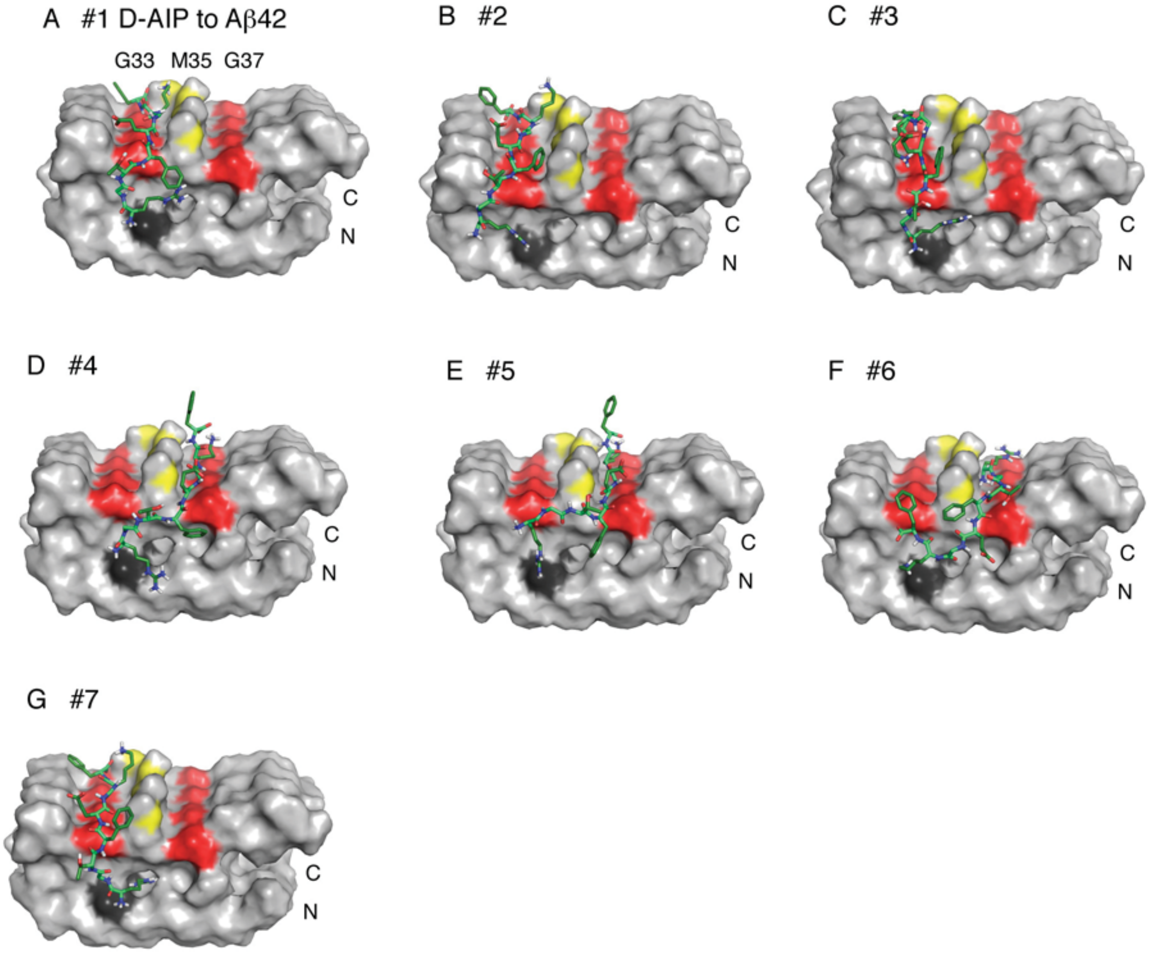

Supplement: Supplementary Information [file srep15410-s1.pdf]
